# Acute exercise modifies titin phosphorylation and increases cardiac myofilament stiffness

**DOI:** 10.3389/fphys.2014.00449

**Published:** 2014-11-20

**Authors:** Anna E. Müller, Matthias Kreiner, Sebastian Kötter, Philipp Lassak, Wilhelm Bloch, Frank Suhr, Martina Krüger

**Affiliations:** ^1^Department of Cardiovascular Physiology, Heinrich Heine University DüsseldorfDüsseldorf, Germany; ^2^Department of Molecular and Cellular Sport Medicine, Institute of Cardiovascular Research and Sport Medicine, German Sport University CologneCologne, Germany

**Keywords:** passive tension, cardiac muscle, connectin, skeletal muscle, posttranslational modification, exercise

## Abstract

Titin-based myofilament stiffness is largely modulated by phosphorylation of its elastic I-band regions N2-Bus (decreases passive stiffness, PT) and PEVK (increases PT). Here, we tested the hypothesis that acute exercise changes titin phosphorylation and modifies myofilament stiffness. Adult rats were exercised on a treadmill for 15 min, untrained animals served as controls. Titin phosphorylation was determined by Western blot analysis using phosphospecific antibodies to Ser4099 and Ser4010 in the N2-Bus region (PKG and PKA-dependent. respectively), and to Ser11878 and Ser 12022 in the PEVK region (PKCα and CaMKIIδ-dependent, respectively). Passive tension was determined by step-wise stretching of isolated skinned cardiomyocytes to sarcomere length (SL) ranging from 1.9 to 2.4 μm and showed a significantly increased PT from exercised samples, compared to controls. In cardiac samples titin N2-Bus phosphorylation was significantly decreased by 40% at Ser4099, however, no significant changes were observed at Ser4010. PEVK phosphorylation at Ser11878 was significantly increased, which is probably mediated by the observed exercise-induced increase in PKCα activity. Interestingly, relative phosphorylation of Ser12022 was substantially decreased in the exercised samples. Surprisingly, in skeletal samples from acutely exercised animals we detected a significant decrease in PEVK phosphorylation at Ser11878 and an increase in Ser12022 phosphorylation; however, PKCα activity remained unchanged. In summary, our data show that a single exercise bout of 15 min affects titin domain phosphorylation and titin-based myocyte stiffness with obviously divergent effects in cardiac and skeletal muscle tissues. The observed changes in titin stiffness could play an important role in adapting the passive and active properties of the myocardium and the skeletal muscle to increased physical activity.

## Introduction

The beneficial effect of regular physical activity on the cardiovascular system has been generally accepted. Not only in healthy humans, but particularly in the setting of cardiovascular diseases, such as heart failure (HF), regular exercise training has been shown to improve cardiac outcome (reviewed in Leosco et al., 2013). HF or impaired cardiovascular reserves in aging subjects are often associated with cardiac β-adrenergic receptor (β-AR) dysfunction and over-activity of the sympathetic nervous system (SNS) (Bristow et al., 1986). According to the current hypotheses regular exercise at least partly restores the normal SNS activity and thereby improves cardiac function (Leosco et al., 2013). In aged subjects this exercise-induced improvement has been shown to be mediated by an enhanced left ventricular inotropic response to Ca^2+^ (Ehsani et al., 1991; Spina et al., 1998).

Recent evidence suggests that changes in the passive titin-based myofilament stiffness may also contribute to the beneficial effects of physical training. Titin stiffness is closely associated with ventricular function and it has been shown that increased titin compliance improves diastolic function (reviewed in Linke and Hamdani, 2014). In contrast, titin stiffening may support the length-dependent activation involved in the Frank Starling mechanism of the heart, which is responsible for the elevated cardiac output in response to increased preload (Methawasin et al., 2014).

The giant protein titin is expressed in all striated muscles and is encoded by a single titin gene. Differential splicing of this gene is the basis for numerous species- and muscle-specific titin isoforms with molecular weights ranging from 3.0 to 3.7 MDa allowing the protein to span a half sarcomere from the Z-disc to the M-line. Due to its gigantic size, its central position in the sarcomere and its elastic I-band domains titin is a scaffold protein important for sarcomere assembly, and serves as a molecular spring that defines myofilament distensibility (for review see Krüger and Linke, 2011). Skeletal muscles express an isoform type called N2A titin (3.3–3.7 MDa) with many muscle-specific splice variants (Freiburg et al., 2000; Prado et al., 2005). In mammalian heart titin is expressed as two main isoform types: the shorter and stiffer N2B isoform (3.0 MDa), and longer and more compliant N2BA isoforms (3.2–3.7 MDa). Titin-based passive myofilament stiffness is largely determined by the expression ratio of the N2BA and N2B titin isoforms (Cazorla et al., 2000). In rat heart the short N2B isoform predominates with approximately 90% of total titin, whereas in healthy human heart the expression ratio is about 40% N2BA:60% N2B. In comparison, the different isoform composition results in stiffer myofilaments in rat hearts compared to human hearts (Krüger and Linke, 2011). More dynamically titin stiffness is modulated via posttranslational modification of the elastic I-band regions N2-B and PEVK. To date, more than 28 phosphorylation sites within these regions have been identified by *in vitro* kinase assays or mass spectrometry (Linke and Hamdani, 2014). Among the characterized phosphorylation motifs are Ser4010 (targeted by PKA and ERK1/2) and Ser4099 (targeted by PKG) in the N2-Bus (Krüger et al., 2009; Raskin et al., 2012), and Ser11878 and Ser12022 (targeted by PKCα and CaMKIIδ) in the PEVK region (Hidalgo et al., 2009; Hamdani et al., 2013). Importantly, phosphorylation of the cardiac specific N2-Bus by cAMP- and cGMP-dependent protein kinases PKA and PKG (Yamasaki et al., 2002; Krüger and Linke, 2006; Krüger et al., 2009), and Ca^2+^/calmodulin-dependent protein kinase II δ (CaMKIIδ) decreases titin-based passive myofilament stiffness (Hamdani et al., 2013), whereas phosphorylation of the PEVK domain by Ca^2+^-dependent protein kinase alpha (PKCα) increases it (Hidalgo et al., 2009).

Changes in titin phosphorylation are a critical hallmark of many cardiac diseases (Linke and Hamdani, 2014), and physical exercise is a promising tool to improve cardiac performance (Brenner et al., 2001; Malfatto et al., 2009). This raises the hypothesis that exercise might alter titin properties. In a recent study performed on cardiac tissue from adult mice exercised for a period of 3 weeks significant changes in the posttranslational modification of the two titin domains N2-Bus and PEVK (Hidalgo et al., 2014) were detected. These changes suggest an exercise-induced increase in cardiac titin compliance, which may help diastolic filling and thereby improve cardiac output in the trained animals. In contrast, the changes in titin modification detected in trained skeletal muscles suggest an increase in titin stiffness, which may help to maintain the structural integrity of the exercised muscle tissue (Hidalgo et al., 2014).

To understand titin's posttranslational modifications induced by exercise training, it is important to study titin properties and biochemistry after acute exercise as a stimulus that activates related signaling pathways. In our present study we therefore investigated effects of a single acute exercise bout on posttranslational modification of titin in cardiac as well as skeletal muscle, and made a first attempt to relate the observed changes to altered protein kinase activation. Our results indicate that acute exercise has different effects on titin stiffness than regular exercise, as it rapidly increases titin-based myofilament stiffness and may therefore support the positive inotropic response of the heart to the elevated physical activity.

## Materials and methods

### Animals and exercise regime

Rats were exercised as previously described (Hamann et al., 2013, 2014). Briefly, adult female Sprague Dawley rats were exercised using a treadmill (20 m/min) for a single 15 min level running bout. The group tested for eccentric downhill exercise conducted the running bout on a treadmill with an angle of −20°. All animals were euthanized directly after finishing the training bout. The control groups were not exercised. Muscle samples were dissected from the left ventricle of the heart and the Musculus vastus lateralis (LAT). Samples were deep-frozen in liquid nitrogen immediately after preparation and stored at −80°C until use. Previous tests from our group confirmed that this procedure effectively preserves the phosphorylation status of titin. We tested cardiac tissue samples from 6 control animals and 3 level running animals of the exercised group. For the skeletal muscle samples, 10 control and 6 level running LAT tissue samples were obtained from both groups. All animal experiments were in accordance with the institutional and the national guidelines and regulations. The experimental procedures were approved by the local animal health and care unit.

### Isolation of rat cardiomyocytes and passive force measurements

For isolation of single rat cardiomyocytes, small samples (3–6 mg) were obtained from the left ventricular muscle strips and transferred into relaxing solution (7.8 mM ATP, 20 mM creatine phosphate, 20 mM imidazole, 4 mM EGTA, 12 mM Mg-propionate, 97.6 mM K-propionate, pH 7.0, freshly supplemented with 30 mM 2,3-butanedione monoxime (BDM), 1 mM dithiothreitol (DTT), 1:100 Protease Inhibitor Cocktail (P8340, Sigma), and 1:200 Phosphatase Inhibitor Cocktail (P0044, Sigma). Samples were then repeatedly homogenized with an ultrathurrax at 750 rpm. The myocyte suspension was then centrifuged with 1000 rpm for 3 min., resuspended and permeabilized for 3 min in the relaxing solution additionally supplemented with 3% Triton-X-100. Myocytes were washed in 3–5 centrifugation steps at 4°C and 2000 rpm using relaxing solution. After each centrifugation step the supernatant was discarded and the myocyte pellet was resuspended in fresh relaxing solution without Triton-X-100. The final myocyte suspension was kept on ice until further experimental use.

For passive force measurements a few microliters of the cardiomyocyte suspension were transferred to a cover slip mounted on an inverse phase contrast microscope (Nicon eclipse Ti). One single cell was selected and fixed between a piezoelectric motor and a force transducer (403A, Aurora Scientific) both covered with a mixture (ratio 2:1) of silicone glues (Dow Corning Glue 3140 and 3145-transparent). Cells were then stretched from slack SL (average, 1.9 μm) in five steps to a maximum sarcomere length (SL) of 2.4 μm. In between the stretches a 5 s holding period was performed to wait for stress relaxation. Following the last stretch-hold, cardiomyocytes were released back to slack SL to test for possible shifts of baseline force. During the stretch protocol SL was recorded using an IMPERX (CCD) camera (Aurora Scientific). From the recordings we analyzed the force at the end of each hold period (near steady-state force). Passive forces of each cell were calculated considering their individual length and depth at initial SL before tightening.

### Sodium dodecyl sulfate polyacrylamide gel electrophoresis (SDS-PAGE)

For titin analyses cardiac and skeletal muscle tissue was homogenized in modified Laemmli buffer (Warren et al., 2003) and proteins were separated by agarose-strengthened 2.1% SDS PAGE as previously described (Opitz et al., 2004; Kötter et al., 2013). Similar protein concentrations were used for each sample and protein bands were visualized by Coomassie stain. The gels were scanned using a Fusion SOLO imager (Vilber) and analyzed densitometrically with Multi Gauge V3.2 software. Average titin-isoform composition was calculated from a minimum of *n* = 3 bands per experimental condition. For PKCα expression level analysis, tissue samples were homogenized in modified Laemmli buffer. Standard 10 and 12.5% SDS-PAGE gels were performed according to standard protocols (Krüger et al., 2009).

### Western blot analysis and phosphospecific antibodies

Titin isoforms were separated by agarose-strengthened 2.1% SDS-PAGE, and were analyzed using two different protein concentrations (15 and 20 μg). Proteins were then transferred onto a PVDF-membrane by semi-dry Western blot technique using the Biorad turbo blot system (1.5A for 25 min with 20 V). Transfer efficiency was checked using Coomassie-based PVDF-stain, and only membranes showing good transfer of titin were used for further analysis. Titin phosphorylation status was tested using phospho-site directed antibodies generated for pSer4010 (VRIEEGKpSLRFPC) and pSer4099 (QANLFpSEWLRNID) in the titin N2-Bus region (peptide nomenclature refers to human cardiac titin; UniProtKB: Q8WZ42), and pSer11878 (CEVVLKpSVLRKR) and pSer12022 (KLRPGpSGGEKP) in the PEVK-region. Antibodies to pSer 4010, pSer4099, and Ser11878 had been generated by Eurogentech (Belgium) as previously described (Kötter et al., 2013). Antibodies to pSer12022 were kindly provided by Henk Granzier (University of Arizona) (Hudson et al., 2011). Incubation of primary antibodies was performed overnight at 4°C for primary and for 2 h at room temperature for secondary antibodies. Goat anti-rabbit IgG conjugated with horseradish peroxidase served as secondary antibody. Signal intensity was analyzed densitometrically (Multi Gauge V3.2 and ImageJ). To detect differences in titin phosphorylation levels we determined, for each sample, the signal intensity of phospho-titin and total-titin. The ratio of phospho:total titin was then used to normalize the phosphorylation status of exercised heart samples to that of untrained controls.

For phospho-PKCα and phospho-Phospholamban analysis, proteins were separated by 10 and 15% SDS-PAGE. Similar protein concentrations were used for each sample and a pre-stained protein ladder (Page Ruler # 26616 from Thermo Scientific) was used as a size standard. Proteins were then transferred onto a PVDF-membrane by semi-dry Western blot technique using the Biorad turbo blot system (1.3A for 7 min with 20V). To determine the expression levels antibodies were used against Phospho-PKCα (phospho-T497, Abcam), (Total-) PKCα (Cell Signaling Technology) and Phospho-Phospholamban (phospho-S16, Badrilla). Mouse and anti-rabbit IgG conjugated with horseradish peroxidase served as secondary antibody. All antibodies were diluted in standard TBST solution supplemented with 0.5–3% BSA or 0.5–3% milk powder. Signal intensity was analyzed densitometrically with ImageJ software. The signal intensity for phosphospecific PKCα or Phospholamban-signal was related to the respective PKCα-total or GAPDH-signal. Afterwards the ratio of phospho:total/GAPDH was used to normalize the status of phosphorylation of exercised muscle samples to the control samples.

### Data analysis

Unpaired Student's *t*-test and Mann-Whitney *U*-test was used to test for statistically significant differences. *P*-values <0.05 and *P*_(exact)_ ≤ 0.001 were taken as an indication of significant differences and, if not noted otherwise, are represented in figures by asterisks.

## Results

### Exercise acutely changes cardiac titin phosphorylation

In order to characterize the effects of acute physical exercise on cardiac passive myofilament stiffness we determined the I-band phosphorylation status of titin using phosphosite-directed antibodies to Ser4010 (PKA-targeted) and Ser4099 (PKG-targeted) in the cardiac-specific N2-Bus region, and to Ser11878 and Ser12022 (PKCα and CaMKIIδ-targeted) in the ubiquitously expressed part of the PEVK region.

**Figure 1:**
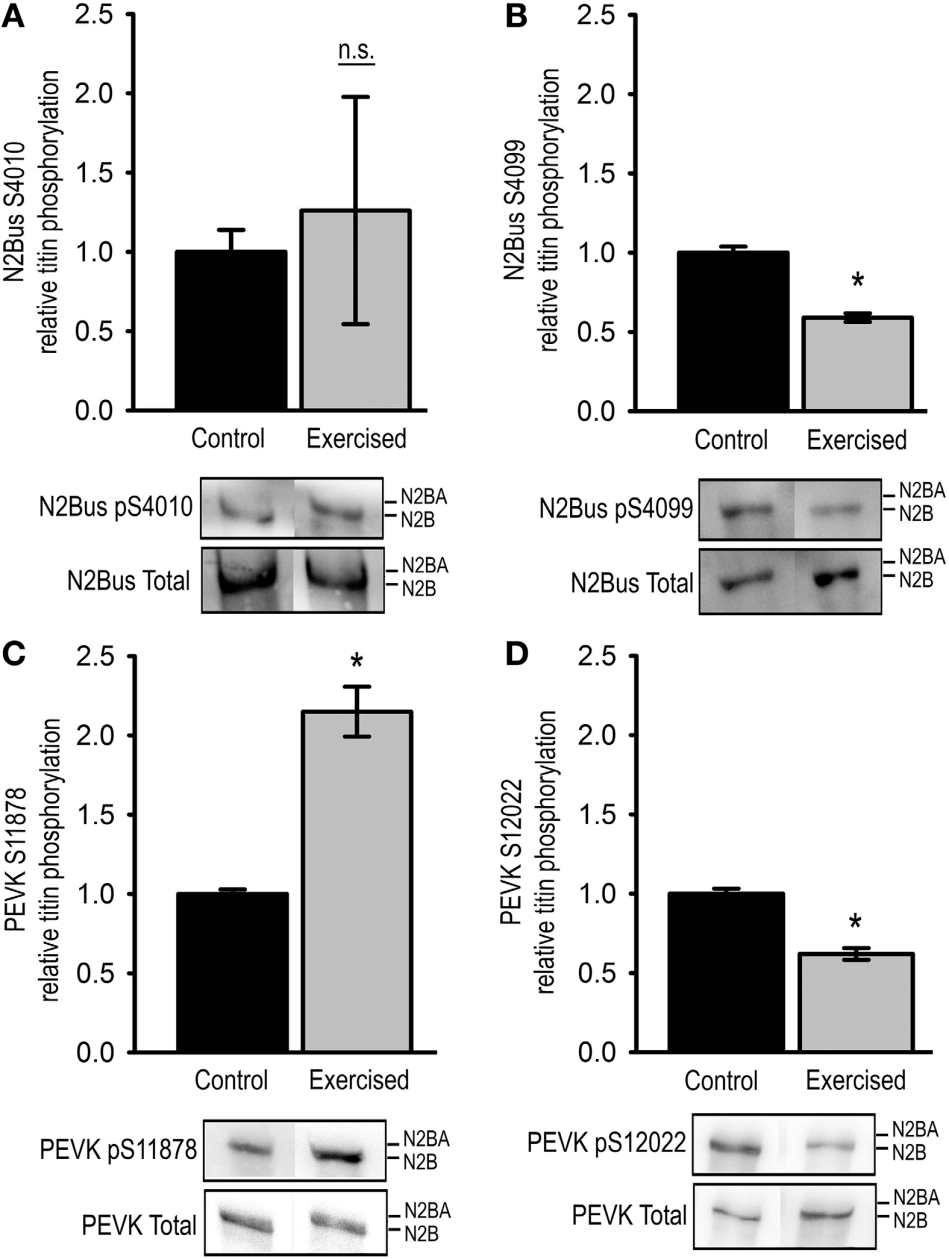
**Titin phosphorylation in cardiac tissue from exercised (Exercised, *n* = 7) and sedentary (Control, *n* = 7) animals**. Bar graphs show statistical data from Western blot analysis using antibodies recognizing **(A)** pSer4010, **(B)** pSer4099, **(C)** pSer11878, or **(D)** pSer12022. Representative images of the Western blots are shown in the lower panel. Graphs show mean ± s.e.m. of a minimum of 3 individual experiments per sample. Asterisks indicate statistically significant differences (*P* < 0.05 in Student's *t*-test and Mann–Whitney *U*-test *P*_(exact)_ = <0.001, n.s. not significant).

As indicated by the large standard errors phosphorylation of residue Ser4010 in the N2-Bus differed largely among the analyzed exercise samples, and with a mean relative phosphorylation of 1.26 ± 0.72 compared to the control group the observed changes were not statistically significantly different (Figure 1A). In contrast, relative phosphorylation of Ser4099 in the N2-Bus was significantly reduced in the cardiac tissue of exercised animals to 0.59 ± 0.03 of the control levels (Figure 1B).

We further tested for changes in phosphorylation at Ser11878 and Ser12022 in the PEVK region of titin. The Western blot analyses demonstrated that relative phosphorylation of Ser11878 was significantly increased in the exercised samples to 2.15 ± 0.16 compared to control samples, whereas relative phosphorylation of Ser12022 was significantly decreased to 0.62 ± 0.04 of the control levels (Figures 1C,D).

### Exercise increases passive cardiac myofilament stiffness

**Figure 2:**
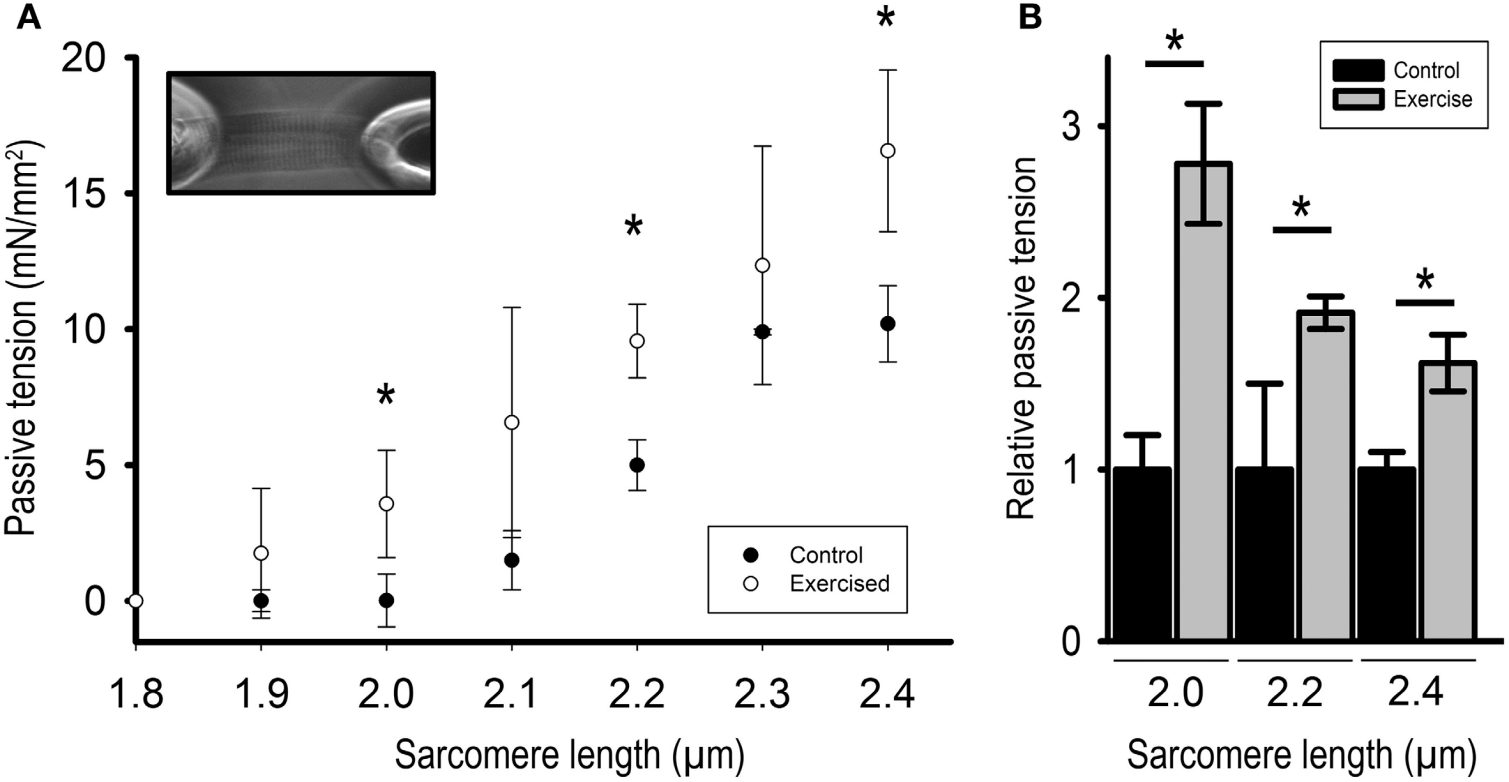
**Passive tension of isolated cardiomyocytes from exercised (Exercised, *n* = 7) and sedentary (Control, *n* = 7) animals in relation to sarcomere length. (A)** Force extension curve showing passive tension (mN/mm^2^) of exercised compared to control cardiomyocytes. Inset: Image of a single cardiomyocyte fixed between a silicon-coated force transducer and a piezo-controlled length driver. **(B)** Bar graphs highlight the statistically significant differences in passive tension at sarcomere lengths (SL) 2.0, 2.2, and 2.4 μm. Graphs show mean ± s.e.m. of a minimum of 3 individual experiments per sample. Asterisks indicate statistically significant differences (^*^P < 0.05 in Student's *t*-test and Mann–Whitney *U*-test *P*_(exact)_ = <0.001).

To determine the effects of altered titin phosphorylation on the passive properties of the cardiac myofilaments we mechanically isolated single cardiomyocytes and measured PT in relation to SL. For this purpose, cardiomyocytes were stretched in five steps to SLs ranging from 1.9 to 2.4 μm and passive forces were recorded for each step. The results demonstrate that PT was significantly higher in cardiomyocytes isolated from acutely exercised animals, compared to controls (Figure 2A). PT was increased 2.78 ± 0.35-fold at SL = 2.0 μm, 1.91 ± 0.10-fold at SL = 2.2 μm, and 1.62 ± 0.17-fold at SL = 2.4 μm compared to cardiomyocytes isolated from sedentary control animals (Figure 2B).

### Exercise acutely modifies skeletal titin phosphorylation

**Figure 3:**
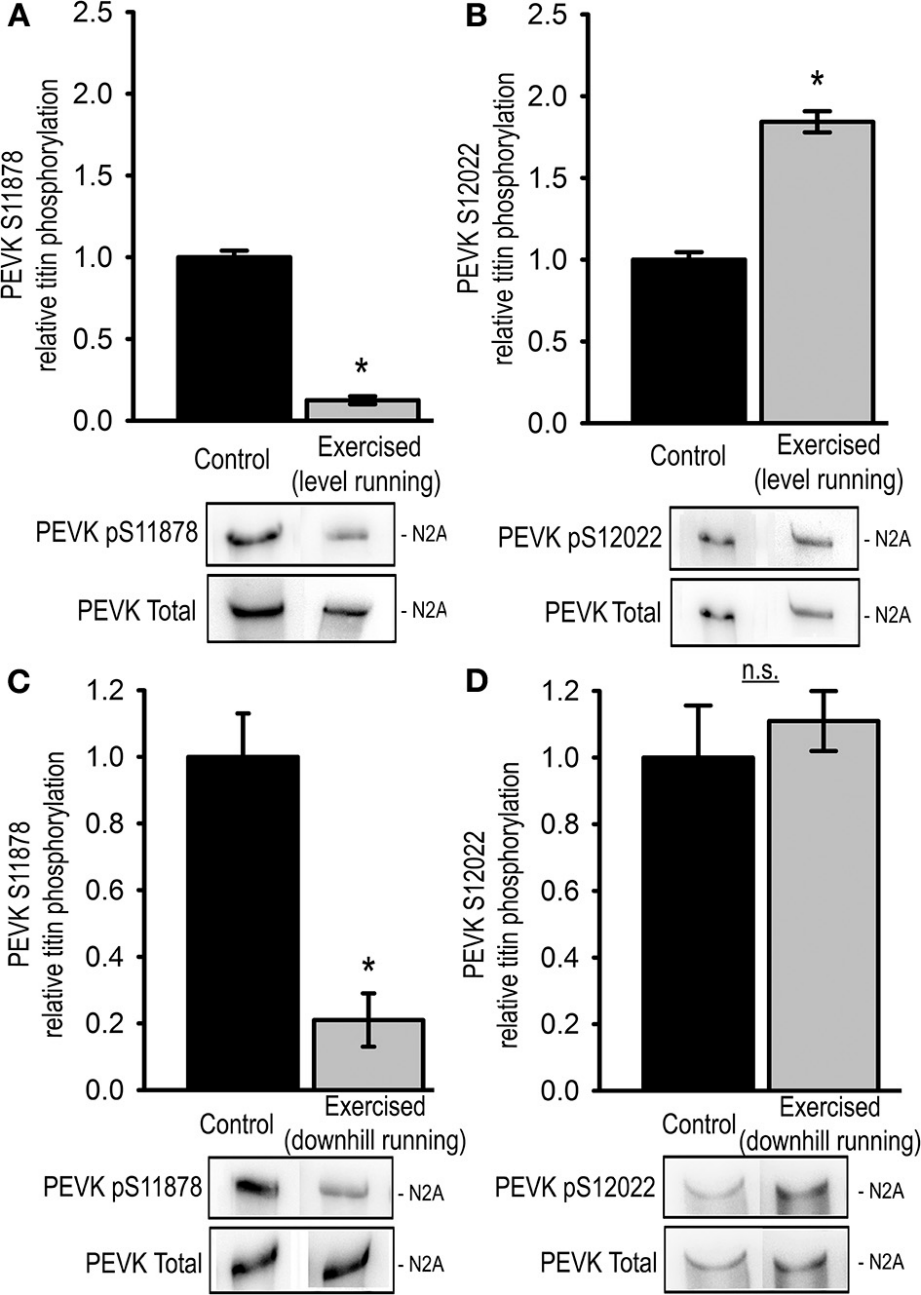
**Exercise-induced changes in skeletal muscle (musculus vastus lateralis) titin phosphorylation**. Bar graphs show statistical data from Western blot analysis from level exercised (Exercised, level running, *n* = 7) and sedentary (Control, *n* = 7) animals using antibodies recognizing **(A)** pSer11878 or **(B)** pSer12022 and from downhill exercised (Exercised, downhill running, *n* = 5) and sedentary (Control, *n* = 5) animals using antibodies recognizing **(C)** pSer11878 or **(D)** pSer12022 in the PEVK region of titin. Bar graphs show mean ± s.e.m. of a minimum of 3 individual experiments per sample. Asterisks indicate statistically significant differences (^*^P < 0.05 in Student's *t*-test and Mann–Whitney *U*-test *P*_(exact)_ = <0.001).

We further investigated the effects of a single exercise bout on skeletal titin phosphorylation by analyzing the relative phosphorylation of Ser11878 and Ser12022 in the PEVK region of titin from the Musculus vastus lateralis (LAT). In order to determine putative differences in the response to concentric and eccentric exercise we analyzed LAT samples from animals after acute level and downhill running, respectively. Interestingly, unlike in cardiac tissue, Western blot analysis of the level-exercised skeletal muscles showed that the relative titin phosphorylation at Ser11878 was significantly reduced to 0.13 ± 0.02 compared to sedentary controls (Figure 3A). At the same time relative phosphorylation of Ser12022 was significantly increased to 1.84 ± 0.06 of the control levels (Figure 3B). Similar results were obtained from LAT muscles of downhill-exercised animals, in which relative titin phosphorylation at Ser11878 was also significantly reduced to 0.21 ± 0.08 (Figure 3C), whereas relative titin phosphorylation at Ser12022 was not significantly changed (Figure 3D).

### Exercise increases PKCα phosphorylation at Thr497 in cardiac but not in skeletal muscle

It has previously been shown that threonine 497 (Thr497) is a critical site for permissive activation of protein kinase Cα (Cazaubon et al., 1994). In a first attempt to investigate the signaling cascades responsible for increased titin PEVK phosphorylation after acute exercise we tested for changes in PKCα activity by determining the relative phosphorylation of Thr497 in cardiac and skeletal samples from exercised and non-exercised animals.

**Figure 4:**
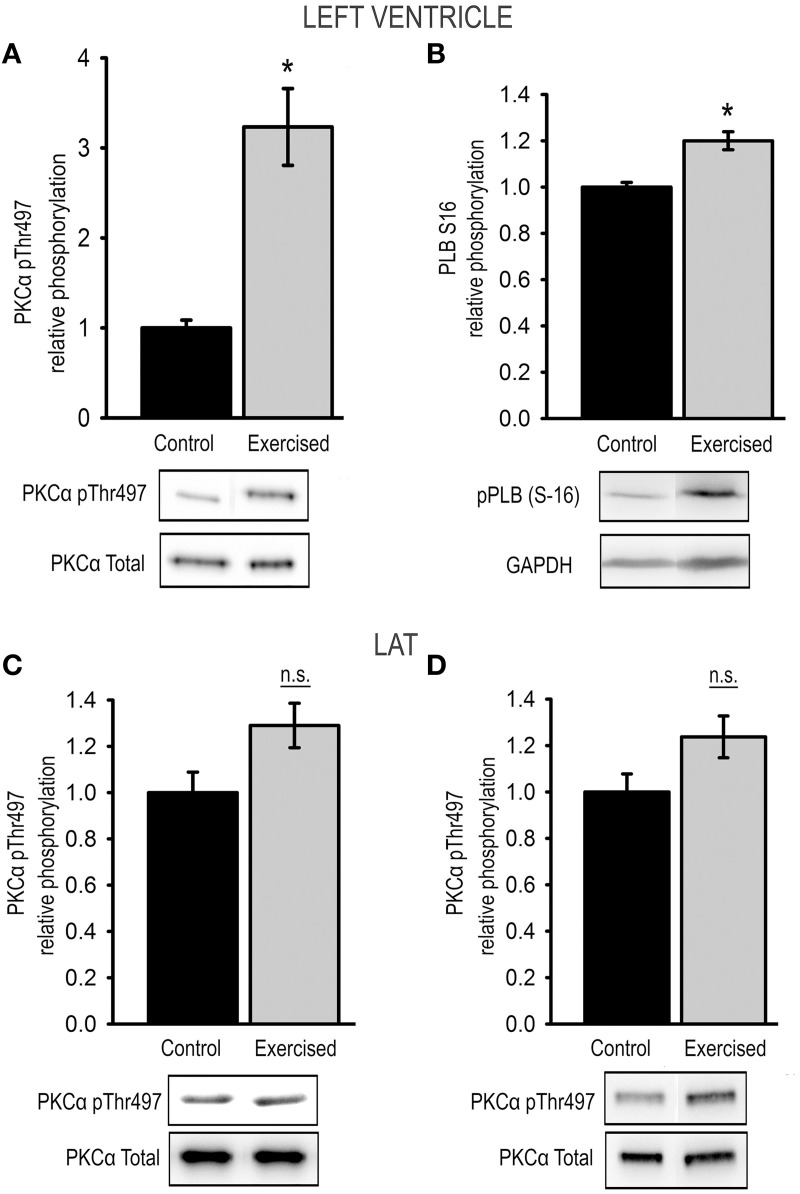
**Exercise-induced changes in kinase activity**. Relative phosphorylation of **(A)** PKCα at Thr497 and **(B)** of the PKA-target phospholamban (pPLB S16) in cardiac muscle tissue from sedentary (control) and exercised (exercised) animals. Relative phosphorylation of PKCα at Thr497 in skeletal LAT muscle tissue from animals after **(C)** level exercise (level running) and **(D)** eccentric downhill exercise (downhill running) compared to sedentary controls (control). Bar graphs show mean ± s.e.m. of a minimum of 3 individual experiments, representative images of the Western blots are shown in the lower panel. Asterisks indicate statistically significant differences (^*^P < 0.05 in Student's *t*-test and Mann–Whitney *U*-test *P*_(exact)_ = <0.001, n.s., not significant).

In cardiac tissue relative Thr497 phosphorylation significantly increased 3.23 ± 0.43-fold in exercised animals compared to sedentary controls (Figure 4A). To test for exercise-induced activation of PKA we determined the relative PKA-mediated phosphorylation of phospholamban (PLB) at position Ser16 using Western blot, and thereby detected a significantly increased activity of PKA in the exercised samples compared to sedentary controls (Figure 4B).

In skeletal muscle tissue relative phosphorylation of Thr497 was not significantly changed with a relative Thr497 phosphorylation of 1.29 ± 0.10 in LAT muscles after level running exercise, and 1.24 ± 0.09 in LAT muscles after downhill exercise compared to sedentary controls (Figures 4C,D).

## Discussion

In our present study we investigated the effects of an acute exercise bout on titin-based myofilament stiffness in cardiac and skeletal muscle tissue from adult rats. The main finding of our study is the observation that in cardiac tissue 15 min. of treadmill running induces a significant increase in passive myocyte tension. Western blot analyses of cardiac samples from acutely exercised rats suggest that this increase is mainly caused by altered titin modification. Our analysis revealed a significant decrease in the relative phosphorylation of residue Ser4099 (PKG-targeted) in the cardiac-specific N2-Bus region, and a significantly increased phosphorylation of residue Ser11878 (PKCα-targeted) in the PEVK-region of titin. Decreased N2-Bus phosphorylation as well as increased PEVK phosphorylation have previously been shown to increase titin-based myofilament stiffness (Hidalgo et al., 2009; Krüger et al., 2009; Kötter et al., 2013). The increased phosphorylation of Ser11878 in response to acute exercise is probably due to a substantial activation of PKCα, indicated by an increased phosphorylation of Thr497 in the activation loop of the kinase (Cazaubon et al., 1994). In this respect, our findings are in accordance with previous reports demonstrating that acute exercise increased cardiac total phospho-PKC epsilon (pSer729), PKC alpha (pSer657) and PKC delta (pThr507) levels (Carson and Korzick, 2003). To date it has been demonstrated that PKCα targets the PEVK-region of titin at Ser11878 and Ser12022 (Hidalgo et al., 2009). Interestingly, in the cardiac samples from exercised animals we observed a significantly lower phosphorylation level of Ser12022 compared to sedentary controls, which is in contrast to the concomitantly observed increase in PKCα activation. However, previous studies suggested that based on differences in the amino acid composition around the PKC phosphorylation motifs within the PEVK region PKCα has a lower affinity to Ser12022 than to Ser11878 (Hidalgo et al., 2009). Since the expression levels of PKCα were not changed within the 15 min. exercise bout the increased activity may be sufficient to increase the phosphorylation status of the high-affinity site Ser11878, but not that of the lower-affinity site Ser12022 (Hidalgo et al., 2014).

Importantly, Ser12022 has also been shown to be targeted by CaMKIIδ, whereas CaMKIIδ-induced phosphorylation of Ser11878 is still under debate (Hamdani et al., 2013; Hidalgo et al., 2013). Hence, the adverse phosphorylation status of the two phosphorylation sites in the PEVK-region may be caused by differences in PKCα as well as CaMKIIδ activity. Nonetheless, PT of single cardiomyocytes was increased in acutely exercised compared to sedentary animals. We therefore assume that the detected decrease in Ser12022 phosphorylation seems to be overruled by the altered titin modification at Ser11878 and at Ser4099 in the N2-Bus region, which is targeted by PKG (Kötter et al., 2013). The reduced phosphorylation of Ser4099 suggests an exercise-induced decrease in ventricular PKG-activity. However, data on cardiac PKG activity in response to acute exercise are not available to date.

It is generally accepted (in humans) that acute exercise is accompanied by activation of the beta-adrenergic system, followed by activation of cAMP-dependent protein kinase A (PKA) (Chasiotis, 1983; Leosco et al., 2013). We therefore determined titin phosphorylation at position Ser4010 in the N2-Bus, which is targeted by PKA (Kötter et al., 2013). Interestingly, relative phosphorylation of this site varied substantially among the analyzed samples and therefore remained statistically unchanged by the 15 min. level exercise bout. To test for exercise-induced activation of PKA we determined the relative PKA-mediated phosphorylation of PLB at position Ser16, and thereby confirmed a significantly increased activity of PKA in the exercised samples compared to sedentary controls. Apparently, under the conditions investigated in our study, this increase does not influence phosphorylation of the PKA-sensitive site Ser4010.

Titin stiffness is closely associated with ventricular function and it is generally accepted that increased titin compliance improves diastolic function (reviewed in Linke and Hamdani, 2014). However, increased titin stiffness may support the length-dependent activation involved in the Frank Starling Mechanism of cardiac muscle (Methawasin et al., 2014), and thus contribute to improved cardiac output in response to acute exercise. To address the question of titin's phosphorylation state in the heart after chronic training, Hidalgo et al. (2014) investigated titin phosphorylation at Ser11878 and Ser12022 in animals trained for a period of 3 weeks and showed significantly decreased Ser12022 phosphorylation, while the phosphorylation of Ser11878 remained unchanged. Based on their results the authors concluded that chronic exercise lowers titin-based stiffness through altered PEVK phosphorylation at Ser12022 and thus improve diastolic function of chronically trained hearts. These data are in contrast to our findings, as we demonstrate acute exercise to increase PEVK-Ser11878 phosphorylation, but to lower PEVK-Ser12022 phosphorylation pointing to increased titin stiffness. Overall, the present results and those from Hidalgo et al. (2014) rise the hypothesis that acute and chronic exercise interventions determine critically different titin phosphorylation states and thus PT in cardiac muscles.

Beside cardiac titin regulation after acute exercise we also focused on skeletal muscle titin regulation in dependence on the primary muscle contraction mode (concentric vs. eccentric). This question is of great interest, since mechanical strains differ between concentric and eccentric muscle work with the consequence of muscle damage specifically after eccentric muscle contractions (Armstrong et al., 1983; Fridén et al., 1984; Fridén and Lieber, 1992). In this context it is known that eccentric exercise has no influence on titin isoform composition in skeletal muscle (Ochi et al., 2007). But surprisingly and due to its central role as an intrasarcomeric mechanosensor in skeletal muscles (Ottenheijm et al., 2011), titin's posttranslational modifications in the PEVK region have never been studied after these two dominant contraction modes. Therefore, we addressed this question and found that both acute concentric and acute eccentric muscle contractions induce similar titin posttranslational modifications, specifically decreased Ser11878 levels in the PEVK region. PKCα activation at Thr497 was unchanged in both concentrically and eccentrically exercised animals compared to sedentary controls. This finding is in contrast to previous reports demonstrating that in skeletal muscle acute exercise bouts increase PKC activity within few minutes after onset of exercise (Rose et al., 2004). However, like in cardiac titin, Ser12022 in skeletal muscle can be phosphorylated also by CaMKIIδ, whose activity had previously been demonstrated to be increased in skeletal muscles upon acute exercise (Rose and Hargreaves, 2003). Therefore, the substantial increase in skeletal muscle titin phosphorylation at Ser12022 after acute running could possibly arise from altered CaMKIIδ activity in the LAT samples. We presume that compared to cardiac tissue the reduced relative phosphorylation of one serine residue (here Ser11878) is likely overruled by the substantial increase in the relative phosphorylation of another serine residue (here Ser12022), and will result in an overall increase in titin-based myofilament stiffness of exercised skeletal muscle tissue. It was surprising to find that, although they induce divergent mechanical strains, acute concentric and eccentric muscle contractions induce similar titin phosphorylation pattern. These data underline that central signaling pathways, such as PKCα, may similarly be regulated by both contraction modes.

An interesting and central finding of our study is that acute exercise induces divergent titin posttranslational modification patterns in cardiac and skeletal muscle tissues. While acute exercise increases titin PEVK phosphorylation at Ser11878 in cardiac muscle, the same intervention results in decreased titin PEVK Ser11878 phosphorylation in skeletal muscle. Opposite effects in cardiac and skeletal muscle could also be detected for titin PEVK Ser12022 phosphorylation. This result could indicate that cardiac and skeletal muscles sense exercise-induced mechanical stress by means of intrasarcomeric proteins, e.g., titin, in a different manner. Our data provide the first direct comparison between cardiac and skeletal muscle titin phosphorylation after a single exercise stimulation. Nonetheless these finding has to be proven by further studies to get a clear picture about titin's regulation in different striated muscle tissues.

We conclude from these observations that acute exercise results in stiffer cardiomyocytes accompanied by increased titin PEVK phosphorylation. For skeletal muscle we postulate that the lowered PEVK phosphorylation leads to a decrease in titin based stiffness. According to this hypothesis, acute exercise induces divergent effects on titin posttranslational modifications and thus myocytes mechanical properties. Considering that the titin spring is a central regulator of the A-band position in the contracting sarcomere, the observed differences in titin stiffness between cardiac and skeletal muscles could be important to maintain their structural integrity, especially of the acutely exercised skeletal muscles For cardiac muscle, our data lead to the assumption that a single exercise bout induces rapid changes in titin-based myofilament stiffness, which may help to improve cardiac output by supporting the Frank-Starling mechanism, possibly crucial for cardiac functions during exercising conditions.

### Conflict of interest statement

The authors declare that the research was conducted in the absence of any commercial or financial relationships that could be construed as a potential conflict of interest.
